# Bucket-Handle Mesenteric Tears: A Comprehensive Review of Their Presentation and Management

**DOI:** 10.7759/cureus.28692

**Published:** 2022-09-02

**Authors:** Ashim Chowdhury, Charlotte Burford, Anang Pangeni, Ashish Shrestha

**Affiliations:** 1 General Surgery, William Harvey Hospital, Ashford, GBR; 2 General and Colorectal Surgery, William Harvey Hospital, Ashford, GBR

**Keywords:** bucket-handle tears, seat-belt injury, abdominal trauma, acute surgical abdomen, mesenteric tears

## Abstract

Bucket-handle mesenteric tears remain a diagnostic challenge for clinicians. We aim to review the literature, including a single-surgeon series, to better understand their presentation and management.

Three electronic databases (Ovid Medline, Embase, and PubMed) were searched for original research articles, describing relevant cases, from database inception to October 2021 using the following Medical Subject Heading (MeSH) terms: mesenteric avulsion, mesenteric tear, and blunt abdominal trauma. A retrospective review of cases managed under a single surgeon at our unit was also performed. Data extracted included demographics, mechanism of injury, presenting features, diagnostic imaging, surgical management, and patient outcome.

In total, 19 studies were identified, including 22 patients (median age 34.5 years). The most common cause of injury was seat-belted road traffic accidents (77.3%), and patients commonly presented with abdominal pain (72.7%), tenderness (50%), positive seat-belt sign (54.5%), and haemodynamic compromise (45.5%). Computerised tomography scanning was the main imaging modality (68%), and the most common findings reported were abdominal free fluid (36.4%) and abdominal wall hernia (27.3%). The majority of patients were operated on within 24 hours of injury (68%), had a median length of stay of 14.5 days, and experienced an uncomplicated recovery (68%). There was no association between the development of complications and delayed surgical intervention >24 hours (p = 0.145). Our institution’s experience was similar, with 50% of patients undergoing surgical intervention within 24 hours. The median age was 32.5 years (50% female), and the median length of stay was 11 days.

A high index of suspicion, serial monitoring, including blood tests, and imaging, with a low threshold for early repeat imaging, can provide a useful guide for identifying patients with bucket-handle tears.

## Introduction and background

A mesenteric bucket-handle tear is a traumatic abdominal injury in which avulsion of the mesentery from a segment of the bowel loop occurs and can result in subsequent de-vascularisation, ischaemia, and hollow-viscus perforation [1]. It has significant associated morbidity and mortality [2] and accounts for the majority of “missed” bowel and mesenteric injuries [3].

Previous studies have suggested that mesenteric or hollow-viscus injuries account for approximately 6% of all blunt abdominal injuries in the United Kingdom [4]. Common features include abdominal pain, bruising, or signs of peritonism [5]. However, the initial presentation can be vague with delayed development of symptoms and signs. Bleeding can be slow and perforation secondary to ischaemia can occur two to three days after the initial injury [6].

Mesenteric bucket-handle tears usually occur because of shearing forces sustained during deceleration injuries, particularly associated with the use of lap seat belts [6], although there is no association between injury severity and the speed of collision. They have also been associated with compression forces arising from bicycle handlebar injuries or from direct force to the abdomen [6]. Areas between fixed and mobile segments of the bowel, such as the ligament of Treitz, are particularly vulnerable to bucket-handle tears, with the majority occurring in the proximal jejunum and the distal ileum near the ileocaecal valve [7].

Computerised tomography (CT) scanning is the most used imaging modality for detecting mesenteric and hollow-viscus injuries in the context of abdominal trauma; however, there are no pathognomonic features [7]. A CT traumagram remains the recommended imaging modality of choice for patients with blunt abdominal trauma despite low sensitivity rates of only 45% [8]. Additionally, the use of a focused assessment with sonography for trauma (FAST) scan is common with a reported sensitivity of around 52% [8]. However, the use of FAST scanning is highly operator-dependent and should only be used in conjunction with other imaging modalities.

A previous systematic review presented 20 cases of mesenteric avulsion following blunt abdominal trauma and found only 25% of cases presenting with shock and/or haemodynamic instability. Previous studies have also suggested that up to 58% of mesenteric avulsion injuries could be missed during the initial clinical assessment and imaging [9].

We ventured to perform this review after four consecutive bucket-handle tear cases, managed in our institution, presented with varying clinical needs and methods of access.

## Review

Methodology

Three electronic databases (Ovid Medline, Embase, and PubMed) were searched for relevant cases from inception to October 2021. The search strategy included the following Medical Subject Heading (MeSH) terms: mesenteric avulsion, mesenteric tear, and blunt abdominal trauma. The search was performed by two separate authors and yielded 1,238 articles, which were screened by title or abstract for relevance.

**Figure 1:**
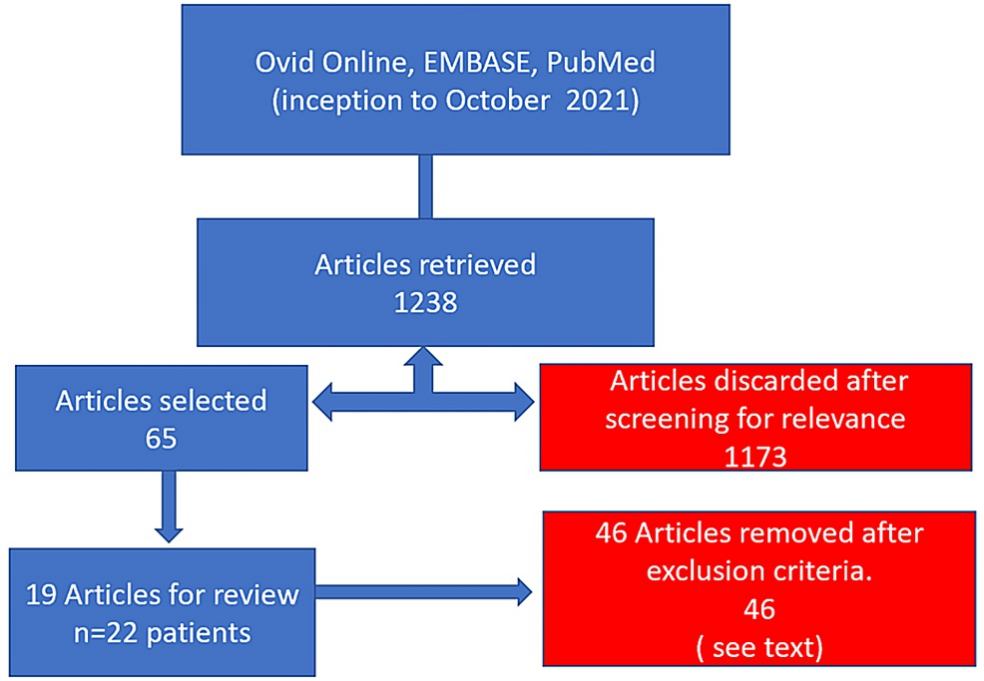
A flowchart illustrating the search strategy and selection of articles for inclusion in this review.

In total, 65 full-text articles were retrieved and individually reviewed. Studies were included if they met the following inclusion criteria: (1) bucket-handle mesenteric injury sustained and confirmed by description or photograph (please note, mesenteric avulsion, mesenteric tear, or mesenteric laceration alone were not sufficient as they do not clarify if the tear was at the mesenteric margin); (2) patient was alive at the time of initial presentation; (3) articles in the English language; and (4) involving human subjects only. Only original research articles, case series, and case reports were included; reviews, systematic reviews, and meta-analyses were excluded. Application of these criteria revealed a total of 19 articles with 22 cases (Figure 1). The references of the 19 articles were also reviewed for any additional cases.

Data extracted included patients’ demographics, mechanism of injury, clinical assessment at presentation (including associated injuries), diagnostic imaging findings, management, time from injury to operative intervention, length of hospital stay, and outcome where available.

A retrospective review of cases managed at our institution at a district general hospital in the South-East of England between January 2017 and June 2021 was also included. Data were collected from electronic patient records and included patients’ demographics, mechanism of injury, clinical assessment at presentation (including associated injuries), diagnostic imaging findings, management, time from injury to operative intervention, length of hospital stay, and outcome where available.

Patient demographics

Most of the cases reported were from North America (n = 10; 47.6%) or Europe (n = 9; 42.9%), with two based in African countries and one based in Australia. Overall, 31.8% of the cohort was female. The average age was 34.5 years (interquartile range (IQR) 21.5-46.75 years), with males being significantly younger than females (32 vs. 56 years. respectively; p = 0.011).

Initial clinical presentation

**Figure 2:**
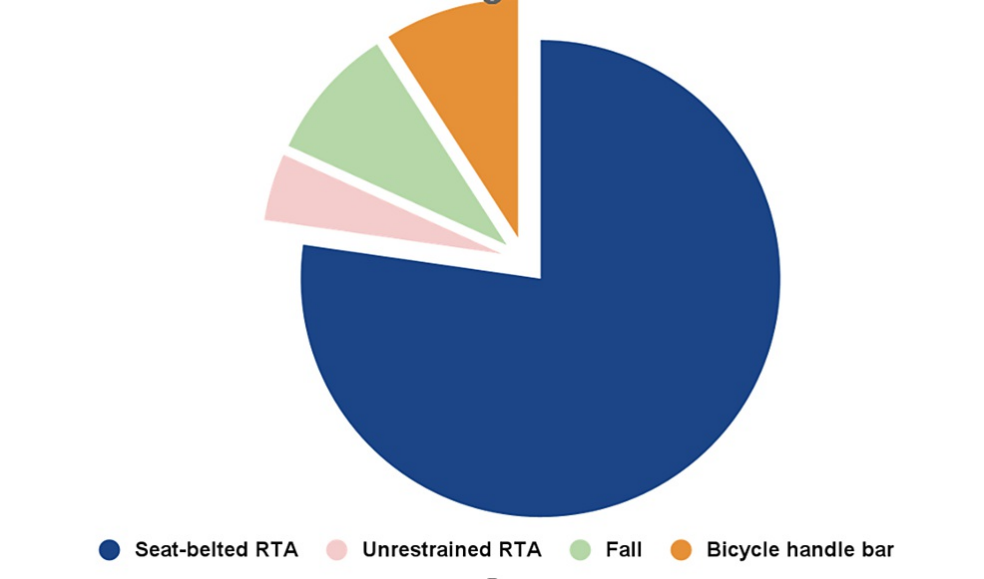
A pie chart showing the proportion of patients presenting with different mechanisms of injury. Overall, 77.3% presented following a seat-belted RTA. RTA: road traffic accident

The most frequent injury mechanism was in seat-belted motor vehicle occupants (n = 17; 77.3%). There was one case of an unrestrained individual who sustained injury secondary from the steering wheel of the vehicle. Two patients presented following a fall, and two suffered a bicycle handlebar injury (Figure 2).

**Figure 3:**
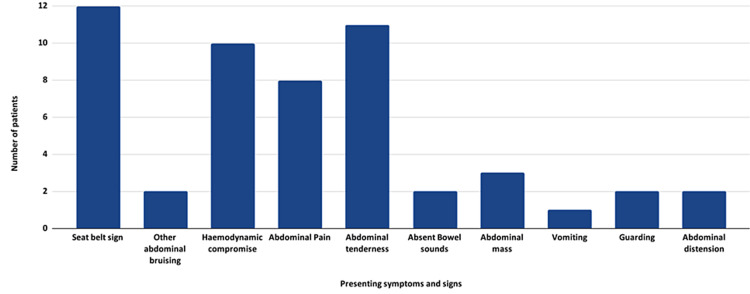
A bar chart showing the number of patients presenting with different symptoms or signs. Other associated injuries, for example, long bone fractures or facial lacerations, are not included.

On arrival at hospital, the most common clinical features were abdominal pain (n = 8; 72.7%) and tenderness (n = 11; 50%) with positive seat-belt sign (n = 12; 54.5%) and haemodynamic instability, including tachycardia (>100 beats per minute) and hypotension (systolic blood pressure <90 mmHg; n = 10; 45.5%) (Figure 3). Two further patients developed signs of haemodynamic compromise later in their admission. Traumatic abdominal wall hernias were present in three (13.6%) patients.

Diagnostic imaging

**Figure 4:**
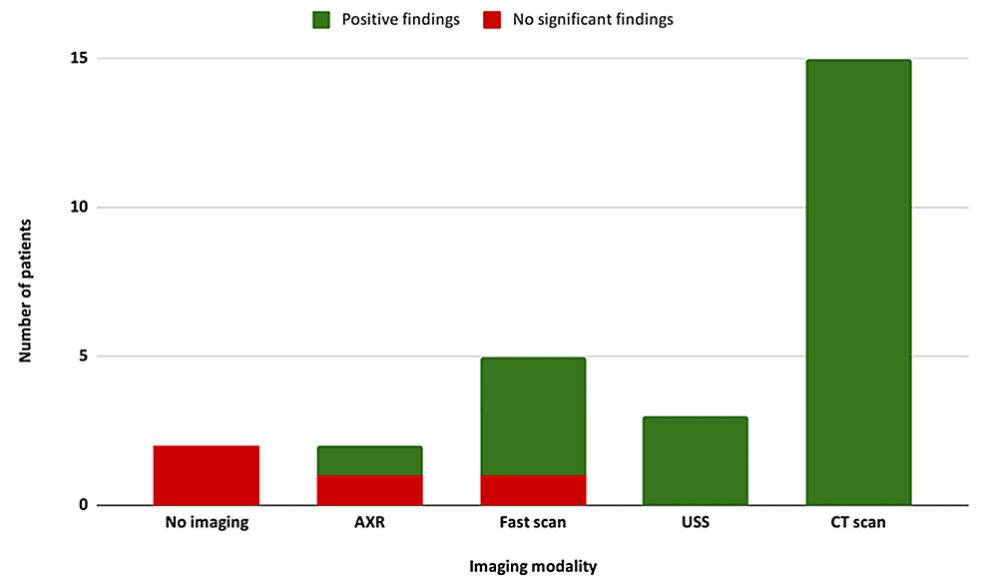
A bar chart showing the different imaging modalities performed and the rate of positive findings (red vs. green).

A total of five patients underwent a FAST scan on admission, with free fluid being demonstrated on all but one (n = 4; 80%). A further three patients underwent abdominal ultrasound with positive findings of free fluid in all three cases (Figure 4).

**Figure 5:**
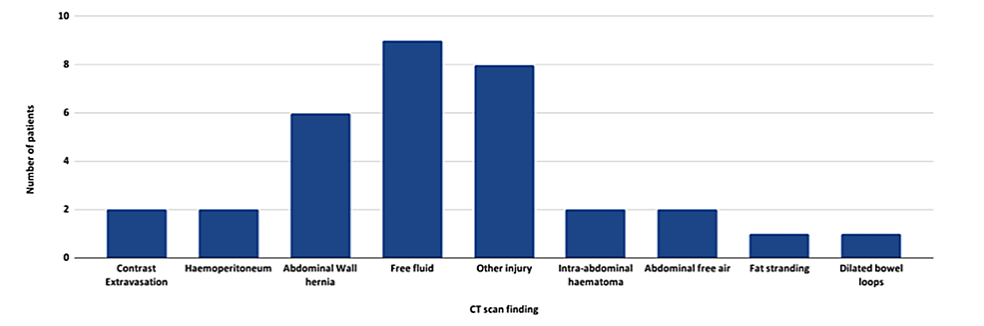
A bar chart showing the key findings on CT scanning. It is important to note these only include the findings reported in the literature and therefore may not include all the features seen in a given individual’s imaging. CT: computerised tomography

CT scan was the main imaging modality, with 15 (68.1%) patients receiving one on admission. In addition, two patients underwent repeat CT scanning during their admission. CT scans with findings warranting emergency surgical intervention were reported in 12 (80%) patients. The most common CT findings reported were abdominal free fluid (n = 8; 36.4%) and abdominal wall hernia (n = 6; 27.3%; Figure 5).

Surgical management

The median time to surgery was 0 days (IQR = 0-1.75 days), with 15 patients undergoing surgery within 24 hours of admission. The longest time to surgery was in a patient who was initially discharged following significant clinical improvement with conservative management. He presented five weeks after the initial presentation with worsening abdominal pain and episodic diarrhoea. He was found to have stenosis of a segment of the small bowel with an associated healing mesenteric bucket-handle tear. Table 1 presents the demographics, clinical features at presentation and the imaging findings for the cases identified in the literature.

**Table 1:** Patient demographics, clinical features at presentation, and imaging findings for the cases identified in the literature. LLQ = left lower quadrant; USS = ultrasound; RLQ = right lower quadrant; FAST = focused assessment with sonography; AXR = abdominal X-ray; L3 = lumber vertebra number 3

Study	Country	Gender/Age	Mechanism of injury	Clinical features at presentation	Imaging
Nosanov et al. (2011) [6]	United States	M/15	Bicycle handlebar injury	Abdominal pain, vomiting, tachycardia, bruising, and localised abdominal tenderness	CT showed free air in the abdomen with LLQ stranding; dilated small bowel loops in LUQ
Kordzadeh et al. (2012) [9]	United Kingdom	F/47	RTA	Seat-belt sign, tachycardia 90 bpm, and paramedian abdominal mass	CT showed a total abdominal wall hernia with small bowel contained
D’Elia et al. (2019) [10]	Canada	F/56	RTA	Abdominal pain, hypotension (95/50 mmHg), and seat-belt sign	CT showed traumatic right flank hernia, trace abdominal free fluid, unspecified orthopaedic injuries
De Backer et al. (1999) [11]	Belgium	M/46	Fall	Mild rebound tenderness and bruising in RLQ; multiple pelvic fractures, and right femoral fracture, right ankle and foot fracture	Minimal free fluid in peritoneum on USS. CT scan confirmed free fluid plus an area of mesenteric haziness and haematoma in RLQ
Doersch et al. (1968) [12]	United States	M/45	RTA	Seat-belt sign, fractured ankle, and multiple facial and head injuries	No details
North et al. (2017) [13]	United Kingdom	M/23	RTA	Initially, no signs or symptoms; re-presented five days after initial discharge with severe abdominal pain	Initial CT showed free fluid in the pelvis
O’Dowd et al. (2011) [14]	Ireland	F/65	RTA	Seat-belt sign, lower abdominal tenderness, right femoral fracture	FAST scan showed free fluid in the right paracolic gutter, pelvis, and around the liver
O’Dowd et al. (2011) [14]	Ireland	F/60	RTA	Seat-belt sign, LUQ tenderness, and guarding, hypotensive (84/47 mmHg)	FAST scan revealed a small amount of free fluid around the liver and spleen. CT scan showed free fluid around the liver and spleen and a large haematoma in the right abdomen with blood in the lesser sac. L1 vertebral fracture was seen
O’Dowd et al. (2011) [14]	Ireland	M/32	RTA	Seat-belt sign; the abdomen was initially soft and non-tender but progressed to acute rigid abdomen	Nil. Haemodynamically unstable so went straight for emergency laparotomy
Shaban et al. (2019) [15]	United States	F/60	RTA	Seat-belt sign and abdominal pain	FAST scan showed free fluid in the pelvis. CT scan showed haemoperitoneum, venous bleeding, posterior lumbar abdominal wall hernia, Chance fracture, and haemopneumothroax
Tonsi et al. (2010) [16]	United Kingdom	M/14	Bicycle handlebar injury	An isolated, tender lump in the right iliac fossa with severe epigastric pain. Guarding and rebound tenderness were observed in the RUQ. Bowel sounds were absent. tachycardic (100 bpm)	CT showed a right abdominal wall defect with small bowel loops protruding into the subcutaneous space. There was free air in the peritoneal cavity and free fluid around the liver and spleen with no solid organ injury
Woo et al. (2009) [17]	United States	M/31	RTA	Abdomen distended but non-tender	CT showed free fluid of unknown origin
Yilmaz et al. (2012) [18]	Turkey	M/32	RTA	Generalized abdominal pain and tenderness in all quadrants. Hypotensive (90/50 mmHg) and tachycardic (110 bpm)	AXR was normal but an abdominal ultrasound revealed diffuse free liquid between the intestinal loops
Sall et al. (2009) [19]	Morocco	M/43	RTA	RIF mass and tenderness. Developed vomiting, fever, and abdominal distension on day three	CT scan showed small bowel abdominal wall hernia
Penningto et al. (2000) [20]	United States	F/18	RTA	Abdominal pain, confusion, hypotensive, tachycardia, and abdominal tenderness	CT scan showed L3 Chance fracture and perihepatic fluid
Voellinger et al. (2011) [21]	United States	M/21	RTA	Seat-belt sign, abdominal tenderness, and mild tachycardia	CT scan showed peritoneal free fluid and T11-12 fractures. Low-density signal in the distal aorta
McCullough et al. (1975) [22]	United Kingdom	M/39	RTA	Abdominal pain with RIF tenderness	-
Hinkley et al. (1954) [23]	United States	F/36	Fall	Abdominal pain, suprapubic bruising, abdominal tenderness, and absent bowel sounds	Dilated small bowel loops without fluid level on AXR

Outcomes

The median length of hospital stay was 14.5 days (IQR = 7-29.75 days). The majority of patients had an uncomplicated recovery (n = 15; 68.1%). Complications included prolonged ileus (n = 2), wound dehiscence of associated mesh repair to abdominal wall hernia site (n = 1), renal failure and sepsis (n = 1), rectus sheath abscess (n = 1), and a pelvic abscess and pleural effusion, both requiring percutaneous drainage (n = 1). There was no association between the development of complications and delayed surgical intervention (>24 hours; Fisher’s exact test, p = 0.145). Table 2 details the management and additional information regarding postoperative recovery for the cases identified in the literature.

**Table 2:** Management details and any additional comments regarding postoperative recovery for the cases identified in the literature.

Reference	Country	Gender/Age	Time to surgical management (days)	Surgical Management	Length of stay (days)	Comments
Nosanov et al. (2011) [6]	United States	M/15	2	Emergency laparotomy with bowel resection and primary anastomosis	7	Uncomplicated recovery
Kordzadeh et al. (2012) [9]	United Kingdom	F/47	0	Emergency laparotomy with primary anastomosis and mesh repair of hernia	7	Uncomplicated recovery
D’Elia et al. (2019) [10]	Canada	M/52	0	Emergency laparotomy with bowel resection and primary anastomosis	-	Uncomplicated recovery
D’Elia et al. (2019) [10]	Canada	F/56	0	Diagnostic laparoscopy converted to laparotomy with bowel resection and primary anastomosis. Tissue repair of traumatic flank hernia	-	Uncomplicated recovery
De Backer et al. (1999) [11]	Belgium	M/46	35	Emergency laparotomy	-	The patient was initially managed conservatively and discharged, re-presented five weeks later with abdominal pain, distention, and episodes of diarrhoea
Doersch et al. (1968) [12]	United States	M/45	0	Emergency laparotomy and bowel resection	-	Uncomplicated recovery
Holland et al. (2000) [24]	Australia	M/13	5	Emergency laparotomy with a defunctioning stoma, later reversed	-	Recovery was complicated by pelvic abscess and a right pleural effusion, both of which were managed with percutaneous drainage
North et al. (2017) [13]	United Kingdom	M/23	5	Emergency laparotomy with bowel resection and primary anastomosis	12	Paralytic ileus
O’Dowd et al. (2011) [14]	Ireland	F/65	0	Emergency laparotomy with bowel resection, side-to-side ileocolic anastamosis, and Hartmann’s procedure	-	Uncomplicated recovery
O’Dowd et al. (2011) [14]	Ireland	F/60	0	Emergency laparotomy with bowel resection and primary anastomosis	60	Postoperative recovery complicated by renal failure and sepsis
O’Dowd et al. (2011) [14]	Ireland	M/32	0	Emergency laparotomy with bowel resection and primary anastomosis	7	Uncomplicated recovery
Shaban et al. (2019) [15]	United States	F/60	0	Emergency laparotomy with bowel resection and primary anastomosis	30	Uncomplicated recovery
Tonsi et al. (2010) [16]	United Kingdom	M/14	0	Emergency laparotomy and bowel resection with primary anastomosis. Suture closure of the musculofascial defect	-	Uncomplicated recovery
Woo et al. (2009) [17]	United States	M/31	0	Emergency laparoscopic resection with primary anastomosis	3	Uncomplicated recovery
Yilmaz et al. (2012) [18]	Turkey	M/32	0	Emergency laparotomy with bowel resection and primary anastomosis	7	Uncomplicated recovery
Sall et al. (2009) [19]	Morocco	M/43	3	Emergency laparotomy with bowel resection and primary anastomosis	20	Uncomplicated recovery
Parrish et al. (2015) [25]	United States	M/12	0	Emergency laparotomy with bowel resection. Colostomy formation on day 3	89	Mesh repair of abdominal wall defect dehisced
Voellinger et al. (2011) [21]	United States	M/21	0	Emergency laparotomy with bowel resection and secondary formation of jejuno-colonic anastomosis after 24 hours	29	Patient also required a distal aortic repair
McCullough et al. (1975) [22]	United Kingdom	M/39	1	Emergency laparotomy with primary anastomosis	17	Prolonged ileus for six days
Hinkley et al. (1954) [23]	United States	F/36	0	Emergency laparotomy with bowel resection	-	Uncomplicated recovery

Institutional case experience

A total of four cases were managed at our institution between January 2017 and June 2021. The median age was 32.5 years, and 50% of the patients were females (n = 2). Three patients presented following road traffic accidents, with one sustaining a handlebar injury from a motorbike during the collision and one presenting following a fall. The clinical features at the time of presentation are shown in Table 3. All four patients underwent a CT traumagram as the initial imaging modality of choice, with two patients having findings requiring urgent surgical intervention. One patient was initially managed conservatively with overnight observation but reported increasing pain, developed haemodynamic compromise with tachycardia and hypotension, and repeated blood tests showed a 15% drop in haemoglobin with rising lactate. A repeat CT scan was performed which showed an increase in abdominal free fluid in the peritoneal cavity. She underwent a delayed laparotomy with small bowel resection and primary anastomosis. Another patient, who was haemodynamically stable at presentation, underwent a diagnostic laparoscopy with peritoneal lavage >24 hours after the initial injury.

**Table 3:** Demographic data, clinical features at presentation, imaging findings, and management approach for the cases managed under a single surgeon at our institution. RTA = road traffic accident; LLQ = left lower quadrant

Age/Gender	Mechanism of injury	Clinical features at presentation	Imaging	Time from presentation to management (days)	Surgical Management	Length of stay (days)	Comments
31y /F	RTA	LLQ tenderness	CT scan showed free fluid in the peritoneal cavity around the liver, spleen, and deep in the pelvis	1	Emergency laparotomy with bowel resection and primary anastomosis	8	Uncomplicated recovery
28y/M	RTA	Mild abdominal pain and bruising over the left aspect of his neck and left chest wall. Tachycardic (120 bpm) with cool peripheries	CT traumogram demonstrated a mesenteric haematoma (5 × 6 × 10 cm) in the LLQ with evidence of contrast extravasation	0	Emergency laparotomy with bowel resection and primary anastomosis	22	Hospital-acquired pneumonia
57y/M	RTA (motorbike handlebar injury)	Mild abdominal pain	CT scan revealed haemorrhagic free fluid around the diaphragm, liver, spleen, and LLQ	0	Emergency laparotomy with ileostomy and mucous fistula formation	11	High-output stoma managed with fluid restriction and loperamide, otherwise uncomplicated
34y/F	Fall	Abdominal pain, loose stool, and shoulder tip pain. Abdominal tenderness	CT showed evidence of haemoperitoneum probably related to small bowel mesentery	2	Diagnostic laparoscopy and peritoneal lavage	6	Uncomplicated recovery

Two patients in our series were managed with resection of de-vascularised bowel segment and primary anastomosis; one patient underwent ileostomy and mucous fistula formation, and the fourth required laparoscopy and peritoneal lavage only. Recovery was complicated by hospital-acquired pneumonia in one patient, who was previously wheelchair-bound due to spastic cerebral palsy associated with severe limb contractures, and by high stoma output in a second patient. The high stoma output was managed conservatively, and the patient was discharged 11 days after admission.

Discussion

Blunt abdominal trauma can result in variable presentations representing a challenge for emergency physicians and trauma surgeons alike. The primary assessment of these patients should follow the routine ABC (airway and cervical spine, breathing, and circulation) approach, with those who are haemodynamically unstable or have signs of peritonitis or frank bleeding proceeding to immediate laparotomy with or without a bedside FAST scan in the emergency department beforehand [10]. Patients who are haemodynamically stable are diagnostically more challenging and should proceed to a CT traumogram to identify any intra-abdominal injuries. While CT is the recommended imaging modality for blunt abdominal trauma, the detection of mesenteric injuries remains difficult, particularly with regard to which mesenteric injuries can be managed conservatively and which require surgical intervention. Bucket-handle mesenteric tears require urgent surgical intervention due to the risk of de-vascularisation and subsequent bowel ischaemia and perforation. A large retrospective study by Extein et al. [7] of CT scans in confirmed bucket-handle injuries reported that free fluid, mesenteric haematomas, and bowel hypo-enhancement were the most frequent findings. In addition, extraperitoneal findings, including Chance fractures and traumatic abdominal wall hernia, should increase the suspicion of an associated hollow viscus injury. This is consistent with the findings reported here, which identified free fluid as the most common finding in patients with bucket-handle tears and abdominal wall hernia as the most common extraperitoneal manifestation on CT scanning. A retrospective analysis by Lannes et al. found that early repeat CT in haemodynamically stable patients could increase the sensitivity for surgically important blunt abdominal injuries from 63.6% to 91.7%. Our institutional experience supports this as planned early repeat CT scanning may have identified increasing peritoneal free fluid in the one case that was initially managed conservatively prior to clinical deterioration. Both the existing literature and our institutional experience highlight the non-specific signs and symptoms seen in patients with bucket-handle tears. Indeed the most common presenting signs and symptoms were abdominal pain, abdominal tenderness, seat-belt sign (bruising), and haemodynamic compromise, which are commonly found in patients with a multitude of injuries secondary to blunt abdominal trauma [26]. In this study, there were six patients (one from our series and five from the literature) who were initially managed conservatively (a further two patients had an extended time between injury and surgery due to delayed presentation to healthcare). Of these six patients, five had imaging done which showed inconclusive features, and all were stable at initial assessment. Four patients deteriorated within 24-72 hours of the initial presentation and proceeded to emergency laparotomy. Two showed significant clinical improvement with conservative management and were discharged from the hospital without surgical intervention. One case re-presented after five days with severe abdominal pain, while the second case re-presented after five weeks with severe abdominal pain and distention and was diagnosed with small bowel obstruction secondary to stricture at the site of the healing bucket-handle tear. Based on these findings, we would recommend that patients with blunt abdominal trauma who are haemodynamically stable, but with non-specific findings on the initial CT scan, should be admitted for at least 48 hours for observation with an early repeat CT scan after 24 hours. Scoring tools may also be useful for making objective assessments of a patient’s clinical condition, allowing rapid decision-making, and avoiding unnecessary surgery or unnecessary delay to surgery. The most well-known scoring tool in the context of blunt abdominal trauma is the Bowel Injury Prediction Score (BIPS). This tool uses clinical (abdominal tenderness), biochemical (white cell count), and imaging (injury grade on CT scan) parameters to predict the presence of a surgically significant injury [27]. The BIPS was created following a retrospective analysis of bowel injuries, scoring patients between 0 and 3. The study reported that patients scoring 2 or more were 19 times more likely to have a surgically significant injury. Subsequent studies have attempted to validate the BIPS tool. One retrospective study of proven bowel injuries found that following the application of the BIPS tool, only 56% of patients would have been identified as having a significant injury [28], while another study reported a positive predictive value of 16%, which would have resulted in a high number of non-therapeutic surgical explorations [29]. However, a recent large study from the United States reported a positive predictive value of 78% using the BIPS tool, with patients who had a BIPS greater than or equal to 2 being 10 times more likely to have a surgically significant bowel or mesenteric injury [30]. Alternative scoring tools have been proposed, for example, one by Raharimanantsoa and colleagues, which also includes injury mechanisms and associated injuries [31]. The score is out of 13, with 8 or greater predicting surgically significant blunt bowel and mesenteric injury, and a positive predictive value of 48% [15]. In the United Kingdom, trauma centres do not currently use scoring tools in blunt abdominal trauma patients routinely, but useful adjunct and prospective databases collecting information from patients with bowel and mesenteric injuries, including bucket-handle tears, could be developed.

## Conclusions

In keeping with other blunt hollow-viscus injuries, bucket-handle mesenteric tears do not have a uniform presentation. Initial clinical features and imaging may provide false reassurance to the clinician; therefore, a high index of suspicion is vital in patients presenting following a motor vehicle collision. Serial observation, blood tests, and imaging, with a low threshold for early repeat imaging, can provide a useful guide for identifying patients requiring surgical exploration. In addition, future studies should continue to seek a valid scoring tool to aid clinical decision-making.
